# An Integrated Network Biology and Molecular Dynamics Approach Identifies CD44 as a Promising Therapeutic Target in Multiple Sclerosis

**DOI:** 10.3390/ph19020254

**Published:** 2026-02-01

**Authors:** Mohammad Abdullah Aljasir

**Affiliations:** Department of Medical Laboratories, College of Applied Medical Sciences, Qassim University, Buraydah 51431, Saudi Arabia; mjasr@qu.edu.sa

**Keywords:** hub biomarkers, Network Analyst, differentially expressed genes, MD simulation

## Abstract

**Background:** Multiple sclerosis (MS) is a neuroinflammatory disease characterized by autoimmune-driven inflammation in the central nervous system that damages axons and destroys myelin. It is difficult to diagnose multiple sclerosis due to its complexity, and different people may react differently to different treatments. While the exact cause of multiple sclerosis (MS) and the reasons for its increasing prevalence remain unclear, it is widely believed that a combination of genetic predisposition and environmental influences plays a significant role. **Methods:** Finding biomarkers for complicated diseases like multiple sclerosis (MS) is made more promising by the emergence of network and system biology technologies. Currently, using tools like Network Analyst to apply network-based gene expression profiling provides a novel approach to finding potential medication targets followed by molecular docking and MD Simulations. **Results:** There were 1200 genes found to be differentially expressed, with CD44 showing the highest degree score of 15, followed by CDC42 and SNAP25 genes, each with a degree score of 14. To explore the regulatory kinases involved in the protein–protein interaction network, we utilized the X2K online tool. The present study examines the binding interactions and the dynamic stability of four ligands (Obeticholic acid, Chlordiazepoxide, Dextromethorphan, and Hyaluronic acid) in the Hyaluronan binding site of the human CD44 receptor using molecular docking and molecular dynamics (MD) simulations. Docking studies demonstrated a significant docking score for Obeticholic acid (−6.3 kcal/mol), underscoring its medicinal potential. MD simulations conducted over a 100 ns period corroborated these results, revealing negligible structural aberrations (RMSD 1.3 Å) and consistent residue flexibility (RMSF 0.7 Å). Comparative examinations of RMSD, RMSF, Rg, and β-factor indicated that Obeticholic acid exhibited enhanced stability and compactness, establishing it as the most promising choice. **Conclusions**: This integrated method underscores the significance of dynamic validations for dependable drug design aimed at CD44 receptor-mediated pathways. Future experimental techniques are anticipated to further hone these findings, which further advance our understanding of putative biomarkers in multiple sclerosis (MS).

## 1. Introduction

Multiple sclerosis (MS) is a chronic, inflammatory, and demyelinating disease of the central nervous system (CNS) that predominantly affects young adults, with a higher prevalence among women [1]. Multiple sclerosis is defined by a complex interaction of genetic susceptibility, environmental influences, and immune system dysfunction, resulting in myelin degradation and axonal injury [2]. The global incidence of multiple sclerosis varies considerably, with estimates between 2 and 150 cases per 100,000 persons yearly, reflecting regional, ethnic, and environmental disparities. The incidence is greatest in temperate climbs, including North America and Northern Europe, and diminished in tropical regions [3]. Multiple sclerosis (MS) presents in several clinical forms, notably relapsing-remitting MS (RRMS), which constitutes over 85% of first diagnoses, alongside progressive variants, including secondary progressive MS (SPMS) and primary progressive MS (PPMS). The variability in illness development highlights the need for individualized strategies in diagnosis and therapy [4]. Genetic study has clarified the hereditary factors involved in the etiology of multiple sclerosis. The human leukocyte antigen (HLA) region, namely the HLA-DRB1*15:01 allele, is significantly correlated with heightened vulnerability to multiple sclerosis (MS) [5]. Genome-wide association studies, or GWASs, have found more than 200 genetic loci linked to multiple sclerosis (MS), involving genes involved in immune modulation, including IL7R, IL2RA, and TNFRSF1A [6]. In addition to these loci, epigenetic processes and gene–environment interactions, such as exposure to Epstein–Barr virus (EBV) and vitamin D insufficiency, further influence the likelihood of developing multiple sclerosis (MS) [7]. At the molecular level, multiple sclerosis pathogenesis is defined by the infiltration of autoreactive T cells, B cells, and macrophages into the central nervous system [8]. This immune response results in the development of inflammatory lesions, demyelination, and axonal degeneration, which are characteristic features of the illness [9]. Genetic mutations affecting immune response regulation and myelin repair are associated with illness severity and progression, providing prospective indicators for prognosis and therapeutic intervention [10]. Therapeutic strategies for multiple sclerosis seek to alter disease progression, diminish relapse frequency, and mitigate symptoms [11]. Initial therapy includes disease-modifying drugs (DMTs) including interferon-β and glatiramer acetate, which regulate immunological function [12]. Recent breakthroughs include monoclonal antibodies that target CD20 (e.g., ocrelizumab) and integrins (e.g., natalizumab), in addition to sphingosine-1-phosphate receptor modulators (e.g., fingolimod). Treatment options for progressive types of MS are restricted; however, high-efficacy disease-modifying therapies and neuroprotective techniques are now being researched [13]. Novel therapeutics concentrate on remyelination and neuroprotection, targeting the fundamental causes of neuronal damage [14]. Experimental methodologies, such as technology for gene editing and transplantation of stem cells, provide potential for the healing of CNS damage and the restoration of function [15]. Moreover, personalized medicine, using genomic and molecular profiling, has the capacity to transform the management of MS according to each patient’s profile [16].

The primary objectives of this work are to identify hub genes and investigate their involvement in the pathophysiology of multiple sclerosis (MS) via the utilization of microarray datasets and analysis using bioinformatics. The project aims to identify crucial regulatory genes involved in MS pathophysiology and examine their connections within biological pathways via the analysis of extensive gene expression data. Furthermore, the research seeks to identify prospective pharmacological targets by merging gene expression data with pharmaceutical databases, therefore paving the way for innovative therapeutic strategies. This study aims to deepen our comprehension of MS and provide a basis for the advancement of precision medicine in its treatment [17].

The ideal hub gene will be used in molecular docking studies, later succeeded by molecular dynamics (MD) simulations to evaluate the effectiveness of the inhibitor against the receptor. These results will enhance future experimental studies as well as the development of novel biomarkers and therapeutic strategies for MS.

Though the analytical methods used in this paper adhere to the well-established bioinformatics workflows, the originality of the work is that the combination of these systems-level layers of differentiation gene expression, protein–protein interaction network topology, upstream kinase-transcription factor regulation, drug-target mapping, and molecular docking with MD simulation are specifically applied to identify CD44-centered molecular signatures in multiple sclerosis. There is no previous research that has combined these studies to the GSE135511 data to emphasize CD44-linked pathways and ligand associations as possible treatment entry points. This combination framework consequently offers fresh biological understanding on top of the individual parts of the analysis.

## 2. Results

### 2.1. PCA and Quality Control Analysis

**Figure 1:**
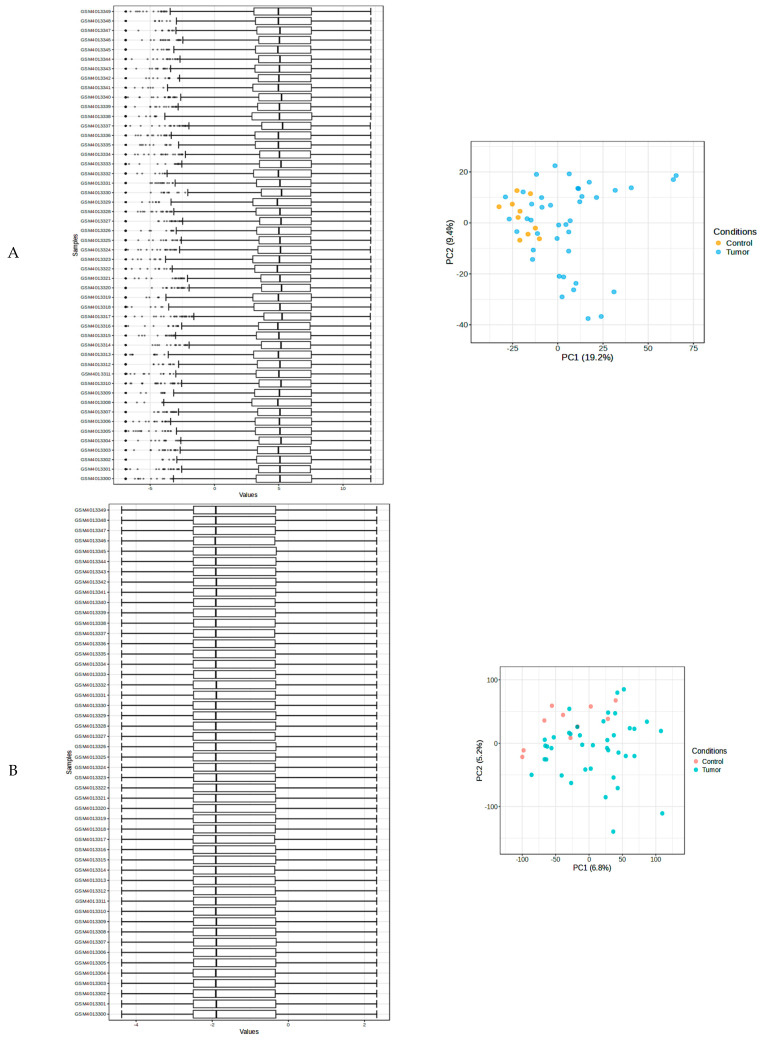
Both boxplots and expression plots were used for the sample visualization, which is shown in the image both before (**A**) and after (**B**) the normalization procedure. Variance stabilization normalization (VSN) and quantile normalization were used to adjust the mean and lower noise.

The information on all individuals was obtained from Table S1 following the analysis of expression profiles employing the MS gene datasets (GSE135511), that were derived from the GEO database of NCBI. Log2FC values and *p*-values obtained from the Network Analyst program were used to discover gene expression differences in both cohorts. DEGs were defined as genes that met the cutoff criteria of *p* < 0.05. The transcriptome expressions were estimated using the VSN quantile normalization method. Figure 1A,B display the mean values and boxplots before and after normalization. The graphics demonstrate that normalization ensures uniform mean values across specimens and eliminates variations in the data. The Principal Componential Analysis (PCA) was conducted to assess sample clustering in terms of gene expressions profile. The normalization process has resulted in PC1 (6.8%) and PC2 (5.2%) of the total variance distinguishing between the control (pink) and the MS (blue) samples, and there is evident clustering between the two groups (Figure 1B). This contributes to the validity of the DEGs between the disease and control cohorts.

### 2.2. Differential Expression Genes DEGs and PPI Network Analysis

**Figure 2:**
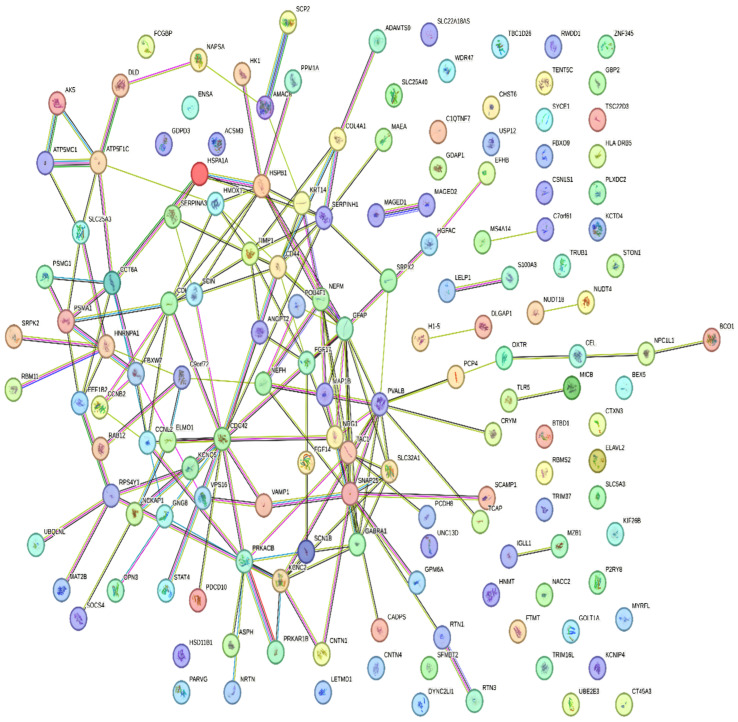
Depicts the network formed by all the DEGs retrieved from the STRING database.

**Figure 3:**
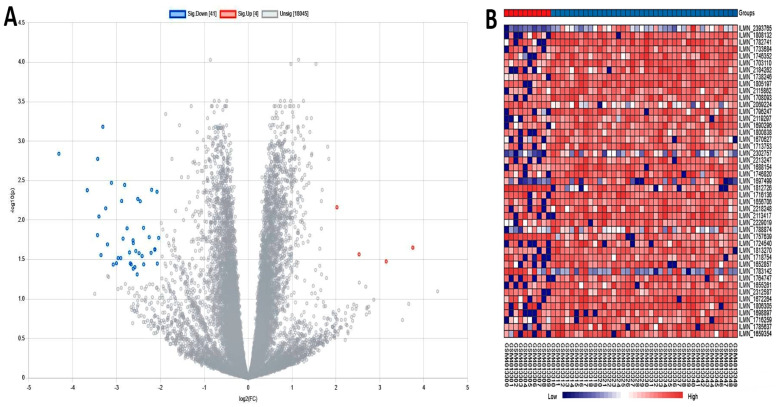
(**A**) A volcano plot displays the intensity distribution of each gene, with red highlighting up-regulated genes, blue highlighting down-regulated genes, and gray highlighting non-significant genes, based on |log_2_ fold change| ≥ 1 and FDR < 0.05. (**B**) Heatmap showing the patterns of the differentially expressed genes in different samples. The expression values were normalized and row-scaled where the low expression was expressed in blue and the high expression in red.

The healthy and sick groups were analyzed using various statistical tests, such as the Benjamini–Hochberg method, the Student t-test, and the Pearson correlation test, which led to the recognition of 45 DEGs. In these DEGs, we identified 4 significant up-regulated and 41 significant down-regulated genes. The final DEGs were ranked according to the adjusted *p*-value. PPI network analysis is a crucial tool for understanding the mechanisms of cellular interaction. By examining the differences in protein networks between healthy and diseased states, we gain insights into the condition’s harshness. This computer model includes representations of both genes and proteins as nodes, with relationships between them shown as edges. A network of interactions for the identified DEGs was created by mapping these proteins and genes. Using Cytoscape, the DEGs were visualized, and interactions between them were retrieved from the STRING database with a medium confidence level of 0.400. The network that all the DEGs create is shown in Figure 2. The network has 169 edges and 150 nodes. It was determined that the mean node degree was 2.25 and the mean local clustering index was 0.364, demonstrating the importance of network connections. The volcano plot (Figure 3A) was plotted based on the significance cutoffs of |log_2_FC| ≥ 1, and FDR < 0.05 distinctly demonstrated significantly up-regulated and down-regulated genes. VST-normalized and row-scaled (Z-score) expression values were used to create the heatmap (Figure 3B) where differential expression patterns of control and MS samples were observed.

### 2.3. Analysis of KEGG Pathway Enrichment and GO Functional Analysis

FDR was used as a critical measure to identify significant keywords used in an examination of enrichment, which included GO cellular components, biological processes molecular functions, and KEGG pathways. Based on the GO cellular component analysis of the DEGs, the proteins were primarily associated with specific granules, tertiary granules, specific granule lumens, tertiary granule lumens, secretory granule lumens, cytoplasmic vesicle lumens, vesicle lumens, and secretory granule membranes. For molecular purposes, two key functions identified for the proteins were carbohydrate binding and lipopolysaccharide binding. Additionally, the enriched biological processes (BPs) associated with the DEGs included the immune response to microorganisms, the humoral immune response, the antimicrobial humoral immune response mediated by antimicrobial peptides, the defensive response, and erythrocyte formation.

The functional enrichment analysis revealed several highly enriched GO terms, KEGG pathways related to immune response and neuroinflammatory signaling. The statistics of enrichment, such as enrichment scores, gene ratios, and FDR-adjusted *p*-values, are summarized in Supplementary Tables S2 (GO) and S3 (KEGG).

### 2.4. Identification of Hub Genes

The hub genes were considered based on node connectivity (degree) to identify the central regulators in the PPI network through the CytoHubba plugin in Cytoscape. The degree values of nodes that are higher than 10 were chosen as hub genes, which contain the largest number of interactions in the network. This cutoff was identified according to the topology of the network, as well as the distribution of node degrees, to obtain only the most generally connected and possibly functionality-important genes that were subjected to downstream analyses. Such kinds of studies have also been established while integrating the computational analysis with experimental approaches to investigate the identified hub genes of the complicated diseases [18,19].

Biologically, hub genes are frequently associated with fundamental proteins that are involved in the coordination of essential cell processes. The nodes that are very closely linked, like CD44, SNAP25, GFAP, PVALB, and HSPB1, are involved in immune modulation, neuronal signaling, and glial cell activity, which are pertinent to disease pathogenesis in the context of MS. For example, CD44, which facilitates leukocyte migration and Hyaluronan signaling, facilitates inflammation and demyelination [20]. On the same note, SNAP 25 is needed to help in the trafficking of synaptic vesicles and the release of neurotransmitters, whereas GFAP and PVALB indicate the activities of the astrocytes and the interneurons, which are disturbed in MS lesions.

**Figure 4:**
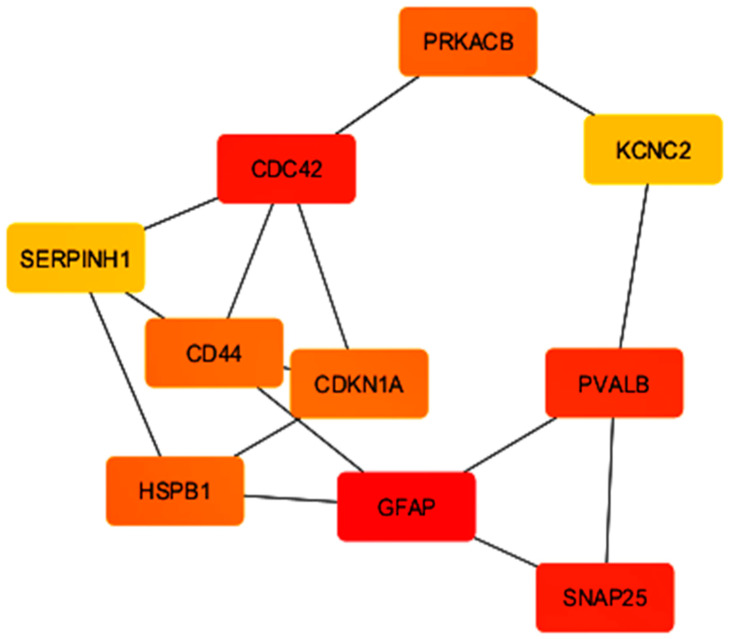
The most direct route interactions for all identified hub genes are displayed, with node colors representing their degree scores.

The top 10 hub genes plus their connectivity scores are tabulated in Table 1. These genes have the shortest path interactions, as shown in Figure 4, and that further demonstrates their central functions in network integrity and their potential applications to MS pathology.

### 2.5. Transcription Factor Analysis

**Figure 5:**
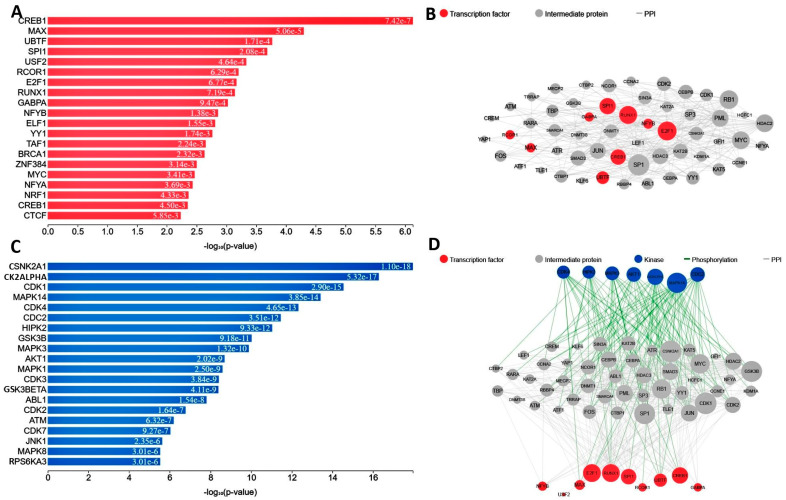
(**A**) The anticipated transcription factors for the DEGs list are displayed, with a bar chart showing hypergeometric scores and *p*-values for each identified transcription factor. (**B**) A ball-and-stick diagram illustrates a sub-network of interconnected transcription factors and their interacting proteins. Pink nodes represent transcription factors, while gray nodes indicate the associated proteins. The size of each node corresponds to its degree within the network. (**C**) A bar graph presents a ranking of the most anticipated kinases, based on their scores derived from hypergeometric *p*-values. (**D**) Illustrating the transcription factors.

This research revealed significant protein kinases and transcription factors associated with the DEGs for their involvement in enhancing the regulated network. A regulatory network was established using transcription factors, kinases, and the transitory proteins to which they were linked throughout the evolution of the regulatory complexity. The first phase was the integration of target genes for transcription factors discovered using assays (ChEA) to forecast the principal transcriptional regulators (TFs), thereafter mapping them into the PPI network. Figure 5A,B illustrate the network and the TFs that were found constructed from PPI analysis. Based on the hypergeometric *p*-value, the key transcriptional regulators in this context include CREB1, MAX, UBTF, SPI1, USF2, and RCOR1. Furthermore, kinases that are likely regulators of the extended PPI networks were identified and mapped onto the PPI network. Figure 5C,D show the key kinases and their connections in the PPI network. Based on the hypergeometric *p*-value, the main kinases linked to these DEGs were identified as CSNK2A1, K2ALPHA, CDK1, MAPK14, CDK4, CDC2, HIPK2, GSK3B, MAPK3, and AKT1.

### 2.6. Hub Genes Survival Investigations

**Figure 6:**
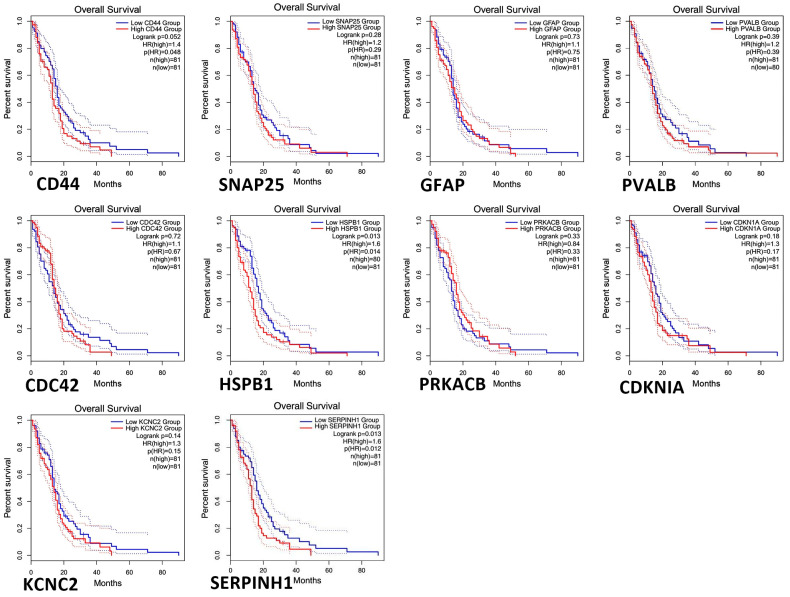
Kaplan–Meier analysis was used to examine the hub genes expressed in patients with multiple sclerosis (MS) to assess overall survival. Survival curves were produced using the Gene-Expression Profiling Interactive Analysis (GEPIA) tool, with statistical significance set at *p* ≤ 0.01. whereas, Differential survival outcomes over time are depicted by the dotted lines that separate the groups with high and low gene expression.

GEPIA survival evaluation was utilized to examine the overall effect on the survival of ten significant hub genes selected from both the down-regulated and up-regulated DEGs. Figure 6 displays the results of the current investigation, with a hazard ratio (HR) of 1.4 and 1.6, respectively. CD44, HSPB1, and SERPINH1 were the only three of the ten genes being studied to exhibit worse overall survival in the group with higher expression.

### 2.7. CD44 and Drug Interactions

Utilizing the best 10 hub genes, prospective pharmaceuticals were discovered from a drug library database. In this investigation, we conducted a manual search for relevant drugs aimed at each hub gene. The query panels included pharmaceuticals from several classifications, namely experimental, FDA-approved, and nutraceuticals. We conducted a thorough evaluation of these target medications to identify those that communicate with each protein designated as a hub gene. A total of 54 pharmacological agents were found for all the hub genes. We have found 1 drug for GFAP, 3 drugs for SNAP25, 3 drugs for CDC42, no drug for PVALB, 10 drugs for CD44, 5 drugs for HSPB1, no drug for PRKACB, 8 drugs for CDKN1A, and 13 for KCNC2. The identified drugs for all these targets are given in Table 2.

### 2.8. Molecular Docking

The principal protein discovered via network-based analysis was subjected to a molecular docking method. The crystal structure of the target protein was obtained from the protein database via PyRx. A blind docking methodology was employed to identify and confirm the precise active site using information derived from the literature. Drug compounds, including Hyaluronic acid, Chlordiazepoxide, Dextromethorphan, and Obeticholic acid, anticipated to interact with the target protein, were formulated based on an examination of the DrugBank database. Hyaluronan (HA) may exhibit proinflammatory functions in the realm of central nervous system autoimmunity. It aggregates in demyelinated lesions of multiple sclerosis (MS), facilitates antigen presentation, and amplifies T cell activation and proliferation [21].

**Figure 7:**
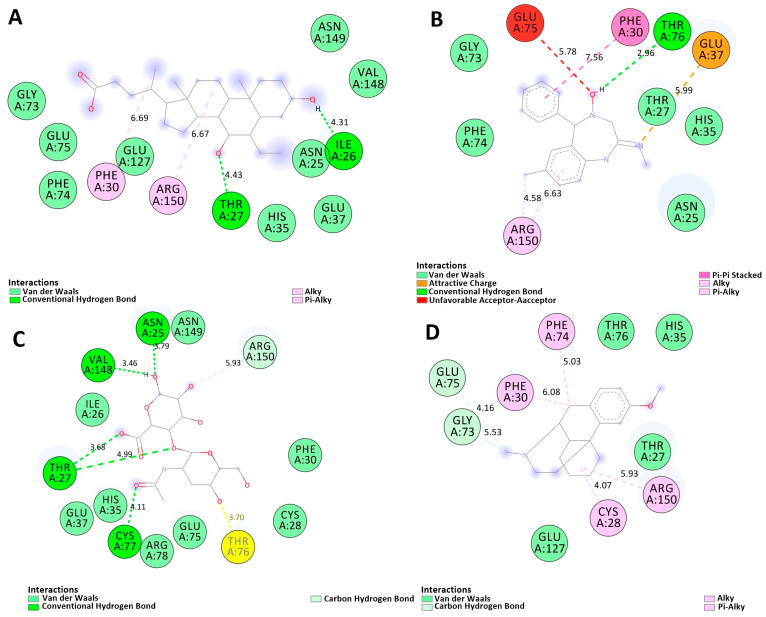
Binding interactions of ligands with the Hyaluronan binding domain of the human CD44 receptor: a comparative analysis of Obeticholic acid (Top1), (**A**), Chlordiazepoxide (Top2), (**B**), Hyaluronic acid shows significant hydrogen bond interactions with active residues and interactions between THR76 and glucose moieties, suggesting a possible glycosylation site or substrate binding interface vital for protein function (Top3), (**C**), and Dextromethorphan (Top4), (**D**) via docking scores and hydrogen bond dynamics.

The results of molecular docking provide essential insights into the efficacy of these medicines for treating multiple sclerosis (MS). MD simulations elucidate the dynamic interactions between ligands and the Hyaluronan binding domain of the human CD44 receptor. This domain pertains to immunological regulation and cell adhesion mechanisms relevant to multiple sclerosis. Under physiological circumstances, Obeticholic acid (Top1) has consistent associations with residues GLU75 and THR76, suggesting its efficacy in inhibiting CD44-mediated pathways, as indicated by a significant docking score of −6.3 kcal/mol (Figure 7A). The stability of Chlordiazepoxide (Top2) is encouraging, as indicated by its interaction with HIS35, suggesting its ability to modulate immunological responses (Figure 7B). The docking score is −6.0 kcal/mol. Dextromethorphan (Top4), with a weak propensity for the binding of −5.3 kcal/mol, may demonstrate variable stability while retaining the therapeutic promise (Figure 7D). Conversely, Hyaluronic acid (Top3) is expected to demonstrate restricted effectiveness in targeting CD44 because of its low docking score (−6.0 kcal/mol), as shown in (Figure 7C). Through the analysis of hydrogen bond dynamics, RMSD, and interaction energies, molecular dynamics simulations substantiate these findings, offering an in-depth understanding of the therapeutic significance and stability of each medication in the context of multiple sclerosis treatment.

**Figure 8:**
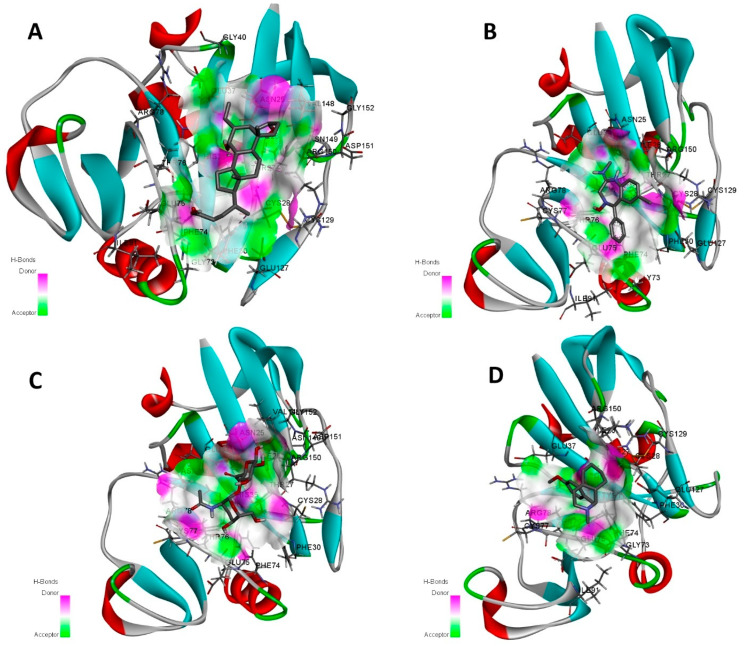
The binding cavity with interactions illustrates hydrogen bond acceptor and donor regions: (**A**) denotes Top1 (Obeticholic acid), (**B**) denotes Top2 (Chlordiazepoxide), (**C**) denotes Top3 (Hyaluronic acid), and (**D**) denotes Top4 (Dextromethorphan), emphasizing the spatial arrangement of donor and acceptor clouds within the Hyaluronan binding domain of the human CD44 receptor. Herein, A distinct binding pocket made of α-helices (red) and β-sheets (blue) holds the ligand. A stable and functionally significant binding mode is supported by these additional structural components, which also allow conserved hydrogen-bond and electrostatic interactions with important active-site residues.

The illustration presents four protein–ligand complexes designated as A, B, C, and D in Figure 8, depicted using ribbon diagrams to emphasize the protein’s secondary structures, including alpha helices (red), beta sheets (cyan), and loops (gray). The ligands are shown in stick format, with binding locations indicated by hydrogen bond donor areas (pink) and acceptor regions (green). In Complex A, the ligand establishes hydrogen bonds with residues GLY40 and ASN25, exhibiting bond lengths of 2.8 Å and 3.1 Å, respectively, signifying robust and stable interactions. Complex B demonstrates the ligand interaction with residues GLU127 and ASP151, exhibiting bond lengths of 3.5 Å and 3.8 Å, indicative of modest binding potential. Complex C exhibits diminished contacts, as the ligand establishes hydrogen bonds with SER50 at 4.2 Å, indicating a weak electrostatic stability. Complex D has moderate binding, characterized by hydrogen bonds between the ligand and residues ARG75 (3.9 Å) and THR100 (3.6 Å), suggesting moderate stability and effectiveness. This visualization highlights the spatial distribution of hydrogen bond donors and acceptors, offering insights into the binding specificity and stability of each ligand inside the protein’s active site (Figure 8).

### 2.9. MD Simulation

**Figure 9:**
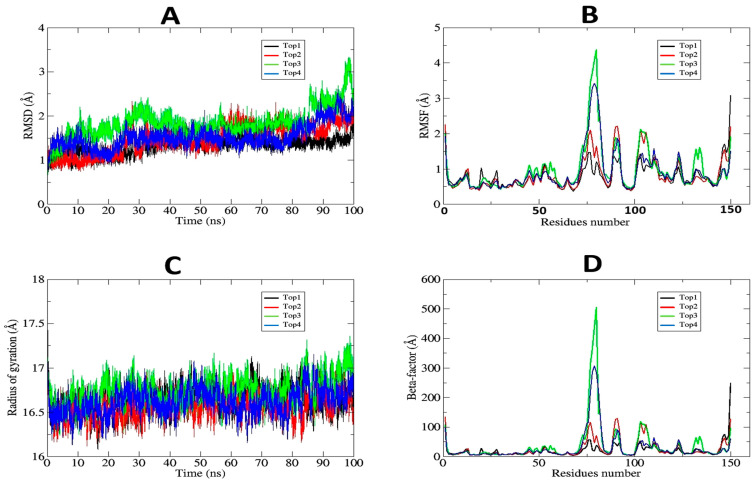
MD simulation results for Top1–Top4 complexes: Panels (**A**,**B**) present RMSF values, indicating residue flexibility; Panel (**C**) illustrates the radius of gyration, demonstrating structural compactness; and Panel (**D**) depicts β-factor analysis, highlighting atomic displacement and dynamic behavior over a 100 ns duration.

The MD simulation findings for the Top1–Top4 complexes provide essential insights into their behavior and stability during interactions with the receptor protein under dynamic settings. Top1 (Obeticholic acid) is recognized as the optimal complex owing to its exceptional stability parameters, with the lowest average RMSD value of 1.3 Å, minimum deviations (maximum RMSD 2 Å, standard deviation 0.18 Å) (Figure 9A), and a compact structure (average Rg 16.6 Å, standard deviation 0.1 Å) (Figure 9C). The data indicate that Top1 preserves structural integrity and stable binding inside the receptor pocket, essential for successful therapeutic activity. Moreover, the low RMSF values (mean 0.7 Å, highest fluctuation 3 Å at residue 149, lowest 0.3 Å at residue 67) signify restricted residue flexibility, hence reinforcing its stability under physiological circumstances (Figure 9B).

In comparison, Top3 (Hyaluronic acid) exhibits the least favorable outcomes, characterized by the greatest average RMSD value (1.9 Å), maximum deviation (3.5 Å), and standard deviation (0.4 Å), indicating considerable variability and diminished binding stability. The elevated RMSF values (mean 0.9 Å, highest fluctuation 4.3 Å at residue 79, minimum 0.4 Å at residue 29) and inconsistent β-factor (mean 37.1 Å, maximum 505.8 Å at residue 79) underscore its diminished dependability and efficacy as a therapeutic candidate (Figure 9D). Top2 (Chlordiazepoxide) and Top4 (Dextromethorphan) exhibit moderate stability, with average RMSDs of 1.5 Å and 1.6 Å, respectively, alongside balanced residue flexibility (mean RMSF of 0.8 Å for both) and compactness (Rg values of 16.5 Å and 16.6 Å, with a standard deviation of 0.1 Å for both), suggesting reasonable yet less robust interactions in comparison to Top1 (Figure 9B,C). Further, the binding free energy calculation predicted all four complexes as stable complexes, where van der Waals forces dominated the overall energy terms, followed by electrostatic energy. The solvation energy, in contrast, contributed non-favorably to complex formation. The net binding energy of Top1, Top2, Top3, and Top4 was estimated as −66.16 kcal/mol, −56.00 kcal/mol, −56.36 kcal/mol, and −49.59 kcal/mol, respectively (Table 3).

Conducting MD simulations after molecular docking is vital for validation and a comprehensive knowledge of ligand–receptor interactions. Docking scores offer a static assessment of binding compatibility but fail to consider dynamic alterations, including conformational changes, flexibility, or stability in real-world settings. MD simulations provide the temporal monitoring of ligand and receptor dynamics, documenting structural deviations (RMSDs), residue fluctuations (RMSFs), compactness (Rg), and atomic displacement (β-factor). These studies facilitate the identification of the most stable and attractive candidates for therapeutic applications, ensuring that computational predictions correspond with practical and physiological relevance. Validation using MD simulations mitigates the likelihood of drug failure in later experimental phases and establishes dependable foundations for drug design and development.

## 3. Discussion

Multiple sclerosis (MS) is a chronic inflammatory disorder that results in demyelination inside the central nervous system (CNS), producing localized lesions in both gray and white matter, as well as extensive neurodegeneration throughout the brain [8]. It is an autoimmune disorder and the primary cause of nontraumatic neurological disability in young adults. It is defined by two key pathological features: (1) inflammation leading to demyelination and (2) astroglial proliferation (gliosis) along with neurodegeneration [12]. MS is influenced by multiple genetic factors that slightly elevate susceptibility, along with well-established environmental contributors such as vitamin D levels, ultraviolet B (UVB) exposure, Epstein–Barr virus (EBV) infection, obesity, and smoking [1]. In recent years, advancements in understanding the mechanisms of RRMS have resulted in the developing of several disease-modifying therapies that help reduce relapse severity and frequency by modulating or suppressing the immune system [15]. Managing MS is challenging and requires multiple drugs with different mechanisms of action, depending on the disease type and progression. While no definitive treatment exists for the primary progressive form, some medications can help alleviate symptoms in the secondary progressive stage and effectively regulate disease activity in relapsing-remitting MS [9].

This research focused on discovering new biomarker candidates and associated protein–protein interaction networks relevant to MS pathways. Transcriptome signature profile data, encompassing microarray and Illumina analysis, were collected from the NCBI GEO database. Gray matter tissues of 50 samples were obtained from multiple sclerosis patients. There were 4 significant up-regulated genes and 41 down-regulated genes. The final DEGs were ranked based on the adjusted *p*-value. The PPI network serves as an essential tool for revealing the structural organization of cellular interactions. GO cellular component (CC) analysis showed that the identified proteins were predominantly localized in the specific granule. The relationship between hub genes and nodes was analyzed and ranked, with red nodes indicating high connectivity. CytoHubba was used to assess node degrees, and those exceeding a degree value of 10 were classified as hub nodes. In addition, significant protein kinases and transcription factors linked to differentially expressed genes (DEGs) were found. The research examined pharmaceuticals from many classifications, including FDA-approved, experimental, and nutraceutical agents. A manual screening process was conducted to identify medications that interacted with each hub gene-associated protein. A total of 54 drugs were identified for each protein. Subsequently, drug molecules were prepared based on predictions from DrugBank database analysis to target the identified proteins. Molecular docking and dynamics simulations were performed, highlighting their significance in elucidating ligand–receptor interactions and assessing the potential therapeutic effectiveness of the examined complexes. The docking data establish a preliminary static framework, elucidating the binding strength and hydrogen bond interactions of the ligands inside the Hyaluronan binding domain of the human CD44 receptor. Among the substances, Obeticholic acid (Top1) had the highest binding affinity, facilitated by significant hydrogen bonds with residues essential for receptor functionality. Conversely, Hyaluronic acid (Top3) had a low binding affinity, indicating its restricted use for medicinal purposes. These findings emphasize the significance of preliminary docking in the identification of lead compounds.

To corroborate and enhance the docking results, molecular dynamics simulations were conducted over a 100 ns period, enabling the observation of real-time ligand–receptor interactions. Top1, aligning with its robust docking score, demonstrated exceptional stability, exhibiting minor structural variations (average RMSD of 1.3 Å) and a compact conformation (average radius of gyration of 16.6 Å). The low RMSF values suggested limited flexibility, demonstrating stable interactions between the ligand and critical residues. Top2 and Top4 exhibited intermediate stability, shown by somewhat elevated RMSD and RMSF values, implying adequate, although less resilient, binding. Conversely, Top3 exhibited considerable variability and instability in both RMSD and RMSF assessments, which corresponded with its diminished docking efficacy. The RMSDs of the complexes can be seen on average to be between 1 and 3 Å, which can be believed as stable dynamics. Some small plot jumps can be seen, which reflect complex loops’ deviation, which are naturally unstable during a dynamic environment, to support proteins’ natural functions [22]. In the RMSFs, the central and N-terminal domains revealed higher local variations compared to the C-terminal domain; however, the net residue fluctuations are well within a stable range. The flexible loops correspond to the unstable fluctuations, which are considerably higher in the central and N-terminal domains. Additionally, the Rg analysis predicted small value changes among the complexes. This illustrates that, as the proteins are the same, the compact nature of the receptor is likely to remain the same, though binding ligands impact the protein compact/relax nature, but in this case, it was observed less.

The amalgamation of the radius of gyration and β-factor investigations yielded more insights into the structural compactness and residue flexibility of the complexes under dynamic settings. Top1 exhibited constant compactness and little atomic displacement, hence reinforcing its potential as a therapeutic candidate. Conversely, Top3 exhibited elevated β-factor values and considerable residue flexibility, underscoring its unstable binding within the receptor pocket.

These findings highlight the need of integrating molecular docking with molecular dynamics simulations to thoroughly assess prospective medicinal medicines. Docking offers a valuable overview of ligand–receptor interactions, whereas molecular dynamics simulations give a dynamic viewpoint, encompassing conformational alterations, binding stability, and residue flexibility. Integrative analyses enhance the trustworthiness of computational predictions, facilitating the identification of stable and effective therapeutic candidates for experimental and clinical investigation. Obeticholic acid (Top1) is identified as the most promising candidate in this study, exhibiting a decent binding profile and consistent interaction dynamics, necessitating additional exploration for therapeutic uses.

Some of the candidate drugs discovered, such as monoclonal antibodies, act on extracellular antigens or cell-surface receptors and may not affect intracellular targets. This limitation clarifies that in silico drug predictions can only be used with caution, given the pharmacological possibility and cellular uptake.

The study is entirely a computational study and no experiments to verify the functional roles of the identified DEGs or the predicted drug candidates were conducted. In silico workflows offer potent funneling insights; however, the inability to determine causal connections between the suggested targets and the pathology of multiple sclerosis is constrained by the lack of in vitro or in vivo validation. Besides that, drug–gene interactions and docking results need additional experimental validation in the form of cell-based assays, molecular characterization, and preclinical models to support their therapeutic potential. Further experiments will be conducted to confirm the biological significance of such computational results through structure and mechanistic methods such as qPCR, Western blotting, and transmission electron microscopy, functional and activity assays (enzyme inhibition assay, cell-based bioassays, flow cytometry, and antimicrobial susceptibility testing), and binding and affinity assays (X-ray crystallography, surface plasmon resonance, isothermal titration calorimetry, etc.).

## 4. Materials and Methods

**Figure 10:**
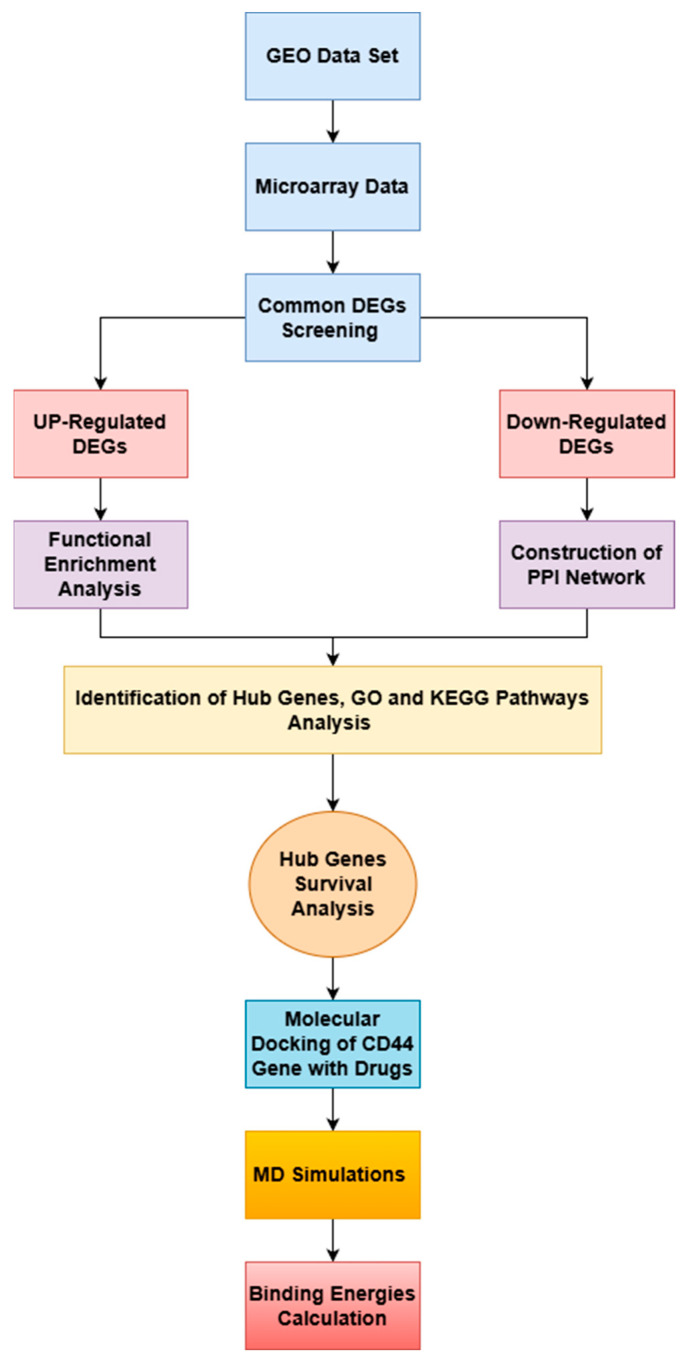
A flowchart showing the thorough bioinformatics analysis method used in this study. The DEGs were identified and subjected to mini up-regulated DEG and down-regulated DEG analyses. The construction of the PPI network for the down-regulated DEGs was performed to ensure the lack of a high-confidence cellular network.

The integrative systems biology analytical framework enables the classification of novel molecular gene pathways, and the identification of unique genetic signatures associated with multiple sclerosis (MS) is shown in Figure 10.

### 4.1. Retrieval Datasets from GEO Database

For this integrated bioinformatics analysis, GSE135511 was retrieved from the NCBI GEO database [23,24]. GEO is an open-access open database that hosts a wide range of high-volume functional genomic information, including microarray studies, next-generation sequencing analyses, and other datasets contributed by research groups worldwide [25]. The GSE135511 database contains 50 samples, of which 10 samples are healthy controls and the other 40 samples have multiple sclerosis; all 50 samples are run on GPL6883 (Illumina HumanRef-8 v3.0 expression beadchip). Gene expression datasets were acquired from GEO datasets. In this study, several analyses were performed, including the identification of sub-networks, sample clustering using principal component analysis (PCA), heatmap visualization of differentially expressed genes (DEGs), construction of the DEG protein–protein interaction (PPI) network, differential expression analysis, identification of hub genes, exploration of drug–protein interaction networks, and molecular docking studies.

### 4.2. Differentially Expressed Gene (DEG) Identification

Network Analyst tool, which is a unified platform for gene expression data analysis, was used to determine and evaluate the genes exhibiting differential expression between normal controls and multiple sclerosis samples, based on data that has statistical significance criteria [26]. The gene set was organized with rows representing individual gene entries and columns corresponding to the specimens. The experimental results indicated an equitable distribution of samples, consisting of 10 controls and 30 sick specimens. Although no further weightings and corrections were made, the normalization and LIMMA analysis were conducted in the same way in all samples. Furthermore, all gene assay identities were correlated with their corresponding Entrez IDs. Network Analyst 3.0 is a sophisticated web-based visual analytics application designed for comprehensive profiling, meta-analysis, and systems-level examination of gene expression data [27]. Each dataset underwent normalization using VSN, log2, and quantile approaches, with normality confirmed by the analysis of box plots and PCA plots. The LIMMA (Linear Models of Microarray Data) workflow of Network Analyst was used to analyze data through a differential expression analysis. The mention of the Student t-test is the moderated t-test in the LIMMA empirical Bayes framework and is not an independent statistical test. LIMMA calculates moderated t-statistics, log ww2-fold changes, and adjusted *p*-values on each gene. To control the false discovery rate (FDR), the Benjamini–Hochberg method was used. Only the exploratory assessment of co-expression was conducted by Pearson correlation analysis, which did not play a role in the determination of the final list of 45 DEGs. Differential gene expressions were considered with FDR less than 0.05 and |log_2_FC| ≥ 1. These thresholds were used to produce volcano plots that could be used to visually depict highly up/down-regulated genes. To visualize the heatmap, the expression matrix was transformed into a normalized expression using the variance-stabilizing transformation (VST) and each gene (each represented as the number of its numeric identifier) within each row was standardized to a Z-score to present the differences in relative expression.

### 4.3. Enrichment and Expression of Genes Analysis

The Network Analyst program enables extensive gene expression profiling and offers support for generic annotation using KEGG orthologs (KOs) [26]. The modules, including differentially expressed genes and core genes, were uploaded with defined parameters to examine functional and pathway enrichment. These included functional mode, gene IDs, species classification such as *Homo sapiens*, molecular functions, biological processes, and pertinent ontologies. Metadata from Gene Ontology (GO) annotations and KEGG pathway databases were used to guarantee a thorough study. Gene Ontology (GO) offers a hierarchical framework for the analysis of gene functions and interactions, inferring functional similarity across genes based on common GO annotations and using information content provided from species data or GO structure [28].

The significance of enrichment was assessed by a hypergeometric test, and the enrichment scores, the ratios of the genes, and adjusted *p*-values were obtained per term. The enrichment outcome of the whole GO and KEGG, along with the enrichment score, the gene ratio, and the FDR-adjusted *p*-value, can be found in Supplementary Tables S1 and S2. To correct the multiple testing, *p*-values obtained after GO and KEGG enrichment analysis were corrected by the Benjamini–Hochberg correction to control the false discovery rate (FDR). Pathways whose FDR was less than 0.05 were deemed to be significant. Herein, the Enrichr server was used to investigate the GO and KEGG with a common technique (Benjamini–Hochberg (BH) approach) for adjusting *p*-values for multiple testing https://maayanlab.cloud/Enrichr/ (accessed on 10 September 2025) [29].

### 4.4. Protein–Protein Interaction (PPI) Network Analysis

The STRING database was used to create and display the protein–protein interaction (PPI) network for differentially expressed genes (DEGs) [30]. The comprehensive database STRING includes both established and predicted protein–protein interactions. These relationships include both direct (physical) and indirect (functional) linkages, which are deduced by computational techniques and forecasts [31]. The preliminary interactions among proteins were shown in Cytoscape 3.10.2 and then assessed for their synergistic effects. Scores must be below 0.75 (showing moderate confidence) to be considered significant. The network configuration was originally restricted to the differentially expressed genes (DEGs), and the full dataset for both DEGs was uploaded. To ensure accurate visualization of the A zero-order interaction method for protein–protein interactions (PPI), networks were selected for the overall network design, which were subsequently constrained to the original DEGs. Cytoscape includes an integrated/built-in “Network Analyzer” tool designed to analyze relationships and interactions within a network of genes and proteins [32].

A medium confidence level STRING score cutoff of 0.400 was chosen in this paper because of its established sensitivity and specificity balance when used to build exploratory networks. Only experimentally studied and database-verifiable interactions were kept, providing sensitivity check with comparison between the network topology at 0.400 and 0.700 thresholds, which revealed stability in identifying the top hub genes (CD44, SNAP25, GFAP). This shows that the selection of hub genes was not contingent on one arbitrary cutoff.

### 4.5. Hub Gene Identification

Cytoscape 3.10.2 was utilized to visualize the protein–protein interaction network derived from the STRING database. This software platform is developed for the visualization, analysis, and interpretation of complex biological systems and molecular interaction information [33]. The nodes with significant degree values were discovered and categorized as hub genes within the network, selected according to their degree metrics. CytoHubba, a Cytoscape plugin, was employed to assess the hub genes within the established PPI network. The ten hub genes exhibiting the shortest pathways were identified as novel therapeutic targets for the treatment of multiple sclerosis.

### 4.6. Correlation of Network of Transcription Factors and Gene Regulators

The correlation between the genes and the regulatory factor was determined using the Expression2Kinase (X2K) program https://maayanlab.cloud/X2K/ (accessed on 10 September 2025). The complete set of DEGs, along with the specified gene markers, was submitted to the X2K software version 1.6.1207 (accessed on 10 September 2025) for analysis. The top ten most important kinases and controls of transcription factors enrichment values were calculated by applying module of kinases and transcription factors (TFs) data. These were constructed from the ChEA69 database and assessed based on Fisher’s exact test *p*-values. X2K Web predicts upstream regulatory networks based on signatures of differentially expressed genes. Through the integration of transcription factors (TFs), kinase enrichment analysis, and PPI network expansion, it generates inferred networks comprising transcription factors, proteins, and kinases that are likely to regulate the expression of the provided gene list. When the network was developed, it was ensured that the edge nodes were properly linked [34].

The ChEA69 database provided the transcription factor–gene interaction via the X2K platform. The curated kinase datasets included in X2K were used to obtain the kinase–gene interactions. The calculated *p*-values based on Fisher’s exact test served as the measure of confidence to rank and select the transcription factors and kinases in the regulatory networks.

### 4.7. Investigation of Targeted Hub Genes Survival

A complete online tool for quick and adaptable gene expression data analysis is the Gene Expression Profiling Interactive Analysis (GEPIA2) platform [35,36]. It provides several analytical characteristics by combining data from the Genotype-Tissue Expression (GTEx) database and The Cancer Genome Atlas (TCGA). GEPIA2 makes it easier to assess which genes show differential expressions, in particular cancer samples, as well as how these genes affect expression patterns and survival outcomes. Through single-gene analysis, the platform was utilized to evaluate the prognostic importance of significant hub genes in the context of (MS), computing the hazard ratio (HR) with a 95% confidence interval and the log-rank *p*-value.

### 4.8. Protein and Drug Interaction Network Construction

To investigate drug–gene interactions, we analyzed the top 10 hub genes. Drug data and target information were retrieved from the latest version of DrugBank Online, which was integrated with Network Analyzer for the analysis. DrugBank includes 500,000+ drugs and their targets. In the latest version of DrugBank 6.0, FDA-approved drugs increased by 72%, increasing from 2646 to 4563. Investigational drugs grew by 38%, from 3394 to 6231. Drug–drug interactions saw a 300% surge, increasing from 365,984 to 1,413,412, while drug–food interactions extended by 200%, from 1195 to 2475 [37]. DrugBank feature entries encompassed approved small-molecule drugs, accepted biologics such as proteins, peptides, allergenics, and vaccines, and nutraceuticals and experimental drugs in the detection phase.

### 4.9. CD44 Molecular Docking with Therapeutics Drugs

The therapeutic target, sourced via the RCSB Protein Data Bank (PDB) having Uniprot ID: P16070, CD44_HUMAN, and PDB ID: 1UUH, had been generated for molecular docking using UCSF Chimera. The docking receptor and ligands were generated using UCSF Chimera, a comprehensive visualization application for structural investigation and energy minimization [38,39,40]. Gasteiger charges were allocated to proteins, and structural limitations were eliminated via 1500 minimization iterations with the ff03.rl force field with a 0.02 step size [41,42]. The reduced protein was confirmed and utilized for docking analyses. The anticipated cavity coordinates were established as center_x = −0.6073, center_y = 1.8726, and center_z = 15.3886, serving as the grid center parameters for the x-, y-, and z-axes in AutoDock Vina version 1.1.2. The grid dimensions were established as size_x = 25, size_y = 25, and size_z = 25 Å, with a spacing of 1.00 Å, guaranteeing exact alignment at the center of the active site. The ligands’ binding affinities were assessed via AutoDock Vina [43]. During the docking process, the most favorable docked compounds were chosen for further analysis utilizing UCSF Chimera [44]. The docking process was validated by considering PDB ID: 4QDI [45], where the co-crystalized ATP molecule was removed from the MurF ligase enzyme and blindly redocked to the protein using the same steps discussed above. The intermolecular conformation of complex after redocking was aligned 3D to the crystalized structure and RMSD was calculated, which was 0.13 Å. The same binding mode of the ATP to the MurF ligase enzyme was reported in both crystalized structures and reported complexes, which implies the accuracy of the docking procedure.

### 4.10. MD Simulation

The dynamic behavior of the docked complex comprising the highest-ranking chemicals was examined using molecular dynamics (MD) simulations. The simulation procedure was executed using AMBER (Assisted Model Building with Energy Refinement), and subsequent analysis was conducted using its various components [46]. The primary coordinates were obtained from the docked complexes, and topology files were generated using the tLEAP interface of AMBER16 [46]. Likewise, tLEAP was employed for the incorporation of absent atoms. Heavy atom side chains of leucine, glutamine, lysine, and aspartate, together with hydrogen atoms, were incorporated into all residue side chains. System solvation was performed using three-point transferable intermolecular potential (TIP3P) water [47], whereas the force fields utilized for calculations comprise GAFF [48] and ff99SB [49]. The docked protein system was neutralized by the addition of 11 Na+ ions, while the ligand atom types and properties were rectified using the antechamber module 17.3. To eliminate steric conflicts, the docked protein complex underwent minimization through 1500 steepest descent steps and 1000 conjugate gradient steps. The system was heated using Langevin dynamics for 10 ps, followed by equilibration for 100 ps in the canonical (NVT) ensemble [50]. During the production run, the SHAKE algorithm was implemented to constrain the lengths of covalent bonds involving hydrogen atoms [51]. The production run lasted a total of 90 nanoseconds. Periodic boundary conditions were employed for all minimization and simulations, with the system size established at a minimum of 8 Å from the protein, while volume, temperature (300 K), and pressure (1 atm) were held constant [46]. The MD production run was performed in triplicate, each time with a different initial velocity, and average structure plots were derived and interpreted. The produced trajectories were examined using the AMBER PTRAJ module [52]. The analysis encompassed Root Mean Square Deviation (RMSD), Beta-Factor (β-factor), Root Mean Square Fluctuation (RMSF), and radius of gyration (Rg) [53]. These post-MD simulation analyses were performed using the CPPTRAJ module of AMBER and are based on carbon alpha atoms [52]. Xmgrace v 5.0 was utilized for graphical demonstration and analytical assessment of these values [54]. The MMGBSA-based binding free energy of complexes was estimated using the MMPBSA.py module of AMBER [55]. The prmtop files were generated through the ante-MMPBSA.py module. In total, 1000 frames were considered from the simulation trajectories at equal time intervals [56].

## 5. Conclusions

In summary, our research highlights the significance of evaluating transcription factors and PPI networks as a sound framework in addition to offering significant new insights into potential biomarkers connected to patient prognoses for multiple sclerosis (MS). Our research primarily aims to identify possible biomarkers and provide a comprehensive understanding of the underlying factors influencing the prognosis of patients with MS. With good binding scores, the molecular docking technique verified all four of the drugs as potential CD44 inhibitors. Also, MD simulations demonstrated the remarkable stability of receptor complexes, such as Top1 and Top3, over 100 ns time in comparison to other complexes, because of structural changes in a physiochemical environment. Remarkably, these inhibitors’ stability was barely affected by minor changes to their side chain and loop mobility. The structural stability seen in the docked complexes following simulation validated the possibility of the selected ligands as lead-like compounds. High binding affinities of the complexes across time intervals were demonstrated by the binding energies that were ultimately computed. Considering these findings, our study urges researchers to examine the development of a customized and precisely targeted medication against MS using the powerful insights gathered from this research. The system’s consistent stability throughout the simulations offers a strong foundation for further study and the creation of effective treatments for multiple sclerosis.

## Figures and Tables

**Table 1:** The top 10 genes from the DEGs network analysis were graded based on data from the STRING interaction database.

Rank	Gene Symbol	Score	Adj. *p*-Value	Log FC
1	CD44	15	0.00206	1.368089
2	SNAP25	14	0.0083515	−1.2745
2	GFAP	14	0.0021351	−1.23569
4	PVALB	13	0.0050701	−2.02376
5	CDC42	9	0.0315718	1.485748
5	HSPB1	9	0.0063439	1.111093
5	PRKACB	9	0.1050604	−0.45806
5	CDKN1A	9	0.0369424	1.079374
9	KCNC2	8	0.0059254	−1.31174
9	SERPINH1	8	0.0053281	1.410022

**Table 2:** Identification of potential drugs targeting specific proteins was conducted through analysis of the DrugBank database.

Sr. No.	Protein	Uniprot ID	Drug Name	DrugBank ID
1	GFAP	P14136	Isopropyl alcohol	DB02325
2	SNAP25	P60880	LetibotulinumtoxinA	DB16820
		P11473	Calcifediol	DB00146
		P12319	Omalizumab	DB00043
3	CDC42	Q96RI1	Obeticholic acid	DB05990
		P10912	Somatrogon	DB14960
		P42345	Everolimus	DB01590
4	PVALB	.	.	.
5	CD44	P20701	Efalizumab	DB00095
		O75330	Hyaluronic acid	DB08818
		Q8N1C3	Chlordiazepoxide	DB00475
		P04234	Muromonab	DB00075
		P11836	Ofatumumab	DB06650
		P11836	Glofitamab	DB16371
		Q8TCU5	Dextromethorphan	DB00514
		Q9NZD1	Talquetamab	DB16678
		P15391	Inebilizumab	DB12530
		Q96RI1	Obeticholic acid	DB05990
6	HSPB1	P35367	Clemastine	DB00283
		P02766	Iodide I-123	DB09420
		P05181	tioconazole	DB01007
		P11473	Dihydrotachysterol	DB01070
		P41143	Tapentadol	DB06204
7	PRKACB	.	.	.
8	CDKN1A	P353637	Clemastine	DB00283
		P11473	Dihydrotachysterol	DB01070
		P23975	Iobenguane	DB06704
		P61073	Motixafortide	DB14939
		P16850	Tioconazole	DB01007
		P08185	Triamcinolone	DB00620
		P35367	Acrivastine	DB09488
		P35367	Azelastine	DB00972
9	KCNC2	P25021	Cimetidine	DB00501
		P41143	Tapentadol	DB06204
		P04150	Clocortolone	DB00838
		P04035	Rosuvastatin	DB01098
		P9Y5N1	Betahistine	DB06698
		P42338	Copanlisib	DB12483
		Q99456	Griseofulvin	DB00400
		P30968	Degarelix	DB06699
		P07550	Salmeterol	DB00938
		P12821	Perindopril	DB00790
		Q9UKR5	Ergosterol	DB04038
		Q14524	Encainide	DB01228
		P23075	Levomilnacipran	DB08918
10	SERPINH1	P35367	Clemastine	DB00283
		P16422	Hypromellose	DB11075
		Q16602	Zavegepant	DB15688
		Q5T9C2	Tamoxifen	DB00675
		P23975	Venlafaxine	DB00285
		P08185	Alclometasone	DB00240
		P31639	Canagliflozin	DB08907
		P23975	Guanethidine	DB01170
		P03372	Estramustine	DB01196

**Table 3:** MMGBSA-based binding free energies. The energies were estimated based on 1000 frames collected at equal intervals of the MD trajectories.

Technique	Energy Section	Top1	Top2	Top3	Top4
MMGBSA	Van der Waals Energy (kcal/mol)	−58.20 (±3.41)	−52.31 (±2.84)	−51.00 (±1.36)	−45.89 (±3.68)
	Electrostatic Energy (kcal/mol)	−14.49 (±2.01)	−12.71 (±2.01)	−13.04 (±1.52)	−11.05 (±1.02)
	Polar Salvation Energy (SE) (kcal/mol)	10.19 (±1.09)	11.50 (±1.36)	10.11 (±1.22)	9.43 (±0.69)
	Non-Polar SE (kcal/mol)	−3.66 (±0.59)	−2.48 (±0.58)	−2.46 (±0.87)	−2.08 (±0.67)
	Gas Phase Energy (kcal/mol)	−72.69 (±4.69)	−65.02 (±3.67)	−64.01 (±4.05)	−56.94 (±3.12)
	Total (kcal/mol)	−66.16 (±4.63)	−56.00 (±3.20)	−56.36 (±3.09)	−49.59 (±2.84)

## Data Availability

The original contributions presented in this study are included in the article/Supplementary Material. Further inquiries can be directed to the corresponding author.

## Supplementary Materials

The following supporting information can be downloaded at: https://www.mdpi.com/article/10.3390/ph19020254/s1, Table S1: Sample information from the GEO database’s GSE135511 datasets. Table S2: Shows the gene of interest involved in GO Biological process. Table S3: Presenting the genes with KEGG enrichment pathways.
