# Supplementary material for: An Integrated Network Biology and Molecular Dynamics Approach Identifies CD44 as a Promising Therapeutic Target in Multiple Sclerosis
Source: Pharmaceuticals (Basel). 2026 Feb 1;19(2):254. doi: 10.3390/ph19020254 (PMC12943740; doi:10.3390/ph19020254)
Supplement: Supplementary file 1 [file pharmaceuticals-19-00254-s001.zip › pharmaceuticals-4087585-supplementary.pdf]

Supplementary files

Table S1. Sample information from the GEO database's GSE135511 datasets.

Group	Accession	Organism	Disease state	Title
**Normal**	GSM4013300	Homo Sapians	Healthy control	Control_Grey_Matter_Tissue_CMS-RZ77
	GSM4013301	Homo Sapians	Healthy control	Control_Grey_Matter_Tissue_CMS-RZ84
	GSM4013302	Homo Sapians	Healthy control	Control_Grey_Matter_Tissue_CMS-RZ90
	GSM4013303	Homo Sapians	Healthy control	Control_Grey_Matter_Tissue_A05-149
	GSM4013304	Homo Sapians	Healthy control	Control_Grey_Matter_Tissue_A05-58
	GSM4013305	Homo Sapians	Healthy control	Control_Grey_Matter_Tissue_A06-189
	GSM4013306	Homo Sapians	Healthy control	Control_Grey_Matter_Tissue_A06-65
	GSM4013307	Homo Sapians	Healthy control	Control_Grey_Matter_Tissue_C14
	GSM4013308	Homo Sapians	Healthy control	Control_Grey_Matter_Tissue_C28
	GSM4013309	Homo Sapians	Healthy control	Control_Grey_Matter_Tissue_C41
**Multiple Sclerosis**	GSM4013310	Homo Sapians	Multiple Sclerosis	GreyMatterLesion_FollicleNegative_MS003
	GSM4013311	Homo Sapians	Multiple Sclerosis	GreyMatterLesion_FollicleNegative_MS042
	GSM4013312	Homo Sapians	Multiple Sclerosis	GreyMatterLesion_FollicleNegative_MS056
	GSM4013313	Homo Sapians	Multiple Sclerosis	GreyMatterLesion_FollicleNegative_MS074
	GSM4013314	Homo Sapians	Multiple Sclerosis	GreyMatterLesion_FollicleNegative_MS100
	GSM4013315	Homo Sapians	Multiple Sclerosis	GreyMatterLesion_FollicleNegative_MS104
	GSM4013316	Homo Sapians	Multiple Sclerosis	GreyMatterLesion_FollicleNegative_MS114
	GSM4013317	Homo Sapians	Multiple Sclerosis	GreyMatterLesion_FollicleNegative_MS127
	GSM4013318	Homo Sapians	Multiple Sclerosis	GreyMatterLesion_FollicleNegative_MS163
	GSM4013319	Homo Sapians	Multiple Sclerosis	GreyMatterLesion_FollicleNegative_MS200
	GSM4013320	Homo Sapians	Multiple Sclerosis	GreyMatterLesion_FolliclePositive_MS079

	GSM4013 321	Homo Sapians	Multiple Sclerosis	GreyMatterLesion_FolliclePositive_MS092
	GSM4013 322	Homo Sapians	Multiple Sclerosis	GreyMatterLesion_FolliclePositive_MS121
	GSM4013 323	Homo Sapians	Multiple Sclerosis	GreyMatterLesion_FolliclePositive_MS136
	GSM4013 324	Homo Sapians	Multiple Sclerosis	GreyMatterLesion_FolliclePositive_MS153
	GSM4013 325	Homo Sapians	Multiple Sclerosis	GreyMatterLesion_FolliclePositive_MS154
	GSM4013 326	Homo Sapians	Multiple Sclerosis	GreyMatterLesion_FolliclePositive_MS160
	GSM4013 327	Homo Sapians	Multiple Sclerosis	GreyMatterLesion_FolliclePositive_MS176
	GSM4013 328	Homo Sapians	Multiple Sclerosis	GreyMatterLesion_FolliclePositive_MS180
	GSM4013 329	Homo Sapians	Multiple Sclerosis	GreyMatterLesion_FolliclePositive_MS234
	GSM4013 330	Homo Sapians	Multiple Sclerosis	Normal Appearing MS Grey Matter_Follicle Negative_MS003
	GSM4013 331	Homo Sapians	Multiple Sclerosis	Normal Appearing MS Grey Matter_Follicle Negative_MS042
	GSM4013 332	Homo Sapians	Multiple Sclerosis	Normal Appearing MS Grey Matter_Follicle Negative_MS056
	GSM4013 333	Homo Sapians	Multiple Sclerosis	Normal Appearing MS Grey Matter_Follicle Negative_MS074
	GSM4013 334	Homo Sapians	Multiple Sclerosis	Normal Appearing MS Grey Matter_Follicle Negative_MS100
	GSM4013 335	Homo Sapians	Multiple Sclerosis	Normal Appearing MS Grey Matter_Follicle Negative_MS104
	GSM4013 336	Homo Sapians	Multiple Sclerosis	Normal Appearing MS Grey Matter_Follicle Negative_MS114
	GSM4013 337	Homo Sapians	Multiple Sclerosis	Normal Appearing MS Grey Matter_Follicle Negative_MS127
	GSM4013 338	Homo Sapians	Multiple Sclerosis	Normal Appearing MS Grey Matter_Follicle Negative_MS163
	GSM4013 339	Homo Sapians	Multiple Sclerosis	Normal Appearing MS Grey Matter_Follicle Negative_MS200
	GSM4013 340	Homo Sapians	Multiple Sclerosis	Normal Appearing MS Grey Matter_Follicle Positive_MS079
	GSM4013 341	Homo Sapians	Multiple Sclerosis	Normal Appearing MS Grey Matter_Follicle Positive_MS092
	GSM4013 342	Homo Sapians	Multiple Sclerosis	Normal Appearing MS Grey Matter_Follicle Positive_MS121
	GSM4013 343	Homo Sapians	Multiple Sclerosis	Normal Appearing MS Grey Matter_Follicle Positive_MS136
	GSM4013 344	Homo Sapians	Multiple Sclerosis	Normal Appearing MS Grey Matter_Follicle Positive_MS153
	GSM4013 345	Homo Sapians	Multiple Sclerosis	Normal Appearing MS Grey Matter_Follicle Positive_MS154

	GSM4013 346	Homo Sapians	Multiple Sclerosis	Normal Appearing MS Grey Matter_Follicle Positive_MS160
	GSM4013 347	Homo Sapians	Multiple Sclerosis	Normal Appearing MS Grey Matter_Follicle Positive_MS176
	GSM4013 348	Homo Sapians	Multiple Sclerosis	Normal Appearing MS Grey Matter_Follicle Positive_MS180
	GSM4013 349	Homo Sapians	Multiple Sclerosis	Normal Appearing MS Grey Matter_Follicle Positive_MS234

Table S2. Shows the gene of interest involved in GO Biological process

Genes	Term	P-value	Adjusted P-value
**CDC42;CD44**	Regulation of Lamellipodium Organization (GO:1902743)	1.48 E-05	0.002858389
**SERPINH1; HSPB1**	Response to Unfolded Protein (GO:0006986)	2.10 E-04	0.020311502
**CDC42;SERPINH1;GFAP**	Supramolecular Fiber Organization (GO:0097435)	5.30 E-04	0.034108876
**CDC42;GFAP**	Regulation of Protein-Containing Complex Assembly (GO:0043254)	0.001714	0.037417734
**HSPB1;CD44**	Cellular Response to Growth Factor Stimulus (GO:0071363)	0.002417	0.037417734
**CDC42**	Positive Regulation of Pinocytosis (GO:0048549)	0.002997	0.037417734
**CDC42**	Establishment of Golgi Localization (GO:0051683)	0.003495	0.037417734
**KCNC2**	Cellular Response to Nitric Oxide (GO:0071732)	0.003495	0.037417734
**KCNC2**	Cellular Response to Reactive Nitrogen Species (GO:1902170)	0.003495	0.037417734
**CD44**	Leukocyte Aggregation (GO:0070486)	0.003495	0.037417734
**GFAP**	Regulation of Chaperone-Mediated Autophagy (GO:1904714)	0.003495	0.037417734
**SNAP25**	Synaptic Vesicle Fusion to Presynaptic Active Zone Membrane (GO:0031629)	0.003994	0.037417734

**PRKACB**	Vascular Endothelial Cell Response to Laminar Fluid Shear Stress (GO:0097700)	0.003994	0.037417734
**CDC42**	Regulation of Pinocytosis (GO:0048548)	0.003994	0.037417734
**SNAP25**	Synaptic Vesicle Docking (GO:0016081)	0.004492	0.037417734
**PRKACB**	High-Density Lipoprotein Particle Assembly (GO:0034380)	0.004492	0.037417734
**KCNC2**	Nitric oxide-cGMP-mediated Signaling (GO:0038060)	0.004492	0.037417734
**CDC42**	Establishment of Epithelial Cell Apical/Basal Polarity (GO:0045198)	0.00499	0.037417734
**CD44**	Regulation of Lamellipodium Morphogenesis (GO:2000392)	0.00499	0.037417734
**KCNC2**	Response to Nitric Oxide (GO:0071731)	0.005488	0.037417734
**CDC42**	Positive Regulation of Pseudopodium Assembly (GO:0031274)	0.005488	0.037417734
**SNAP25**	Neurotransmitter Uptake (GO:0001504)	0.005488	0.037417734
**CDC42**	Regulation of Pseudopodium Assembly (GO:0031272)	0.005488	0.037417734
**HSPB1;GFAP**	Regulation of Autophagy (GO:0010506)	0.005576	0.037417734
**CD44**	- Reg of Intrinsic Apoptotic Sgnlng Pway in Resp to DNA Damage by P53 Cls Mediator (GO:1902166)	0.005985	0.037417734
**PRKACB**	Vascular Endothelial Cell Response to Fluid Shear Stress (GO:0097699)	0.005985	0.037417734
**SNAP25**	Synaptic Vesicle Membrane Organization (GO:0048499)	0.006482	0.037417734
**CD44**	Positive Regulation of Heterotypic Cell-Cell Adhesion (GO:0034116)	0.006482	0.037417734

**CD44**	Reg of Intrinsic Apoptotic Sgnlng Pway in Resp to DNA Damage by P53 Cls Mediator (GO:1902165)	0.006482	0.037417734
**CD44**	Hyaluronan Metabolic Process (GO:0030212)	0.006482	0.037417734
**CDC42**	Regulation of Actomyosin Structure Organization (GO:0110020)	0.006482	0.037417734
**CDC42**	Regulation of Attachment of Spindle Microtubules to Kinetochore (GO:0051988)	0.006482	0.037417734
**CD44**	Epiboly Involved in Wound Healing (GO:0090505)	0.006979	0.037417734
**CDC42**	Establishment of Apical/Basal Cell Polarity (GO:0035089)	0.006979	0.037417734
**CDC42**	Polarized Epithelial Cell Differentiation (GO:0030859)	0.006979	0.037417734
**HSPB1**	Positive Regulation of Endothelial Cell Chemotaxis (GO:2001028)	0.006979	0.037417734
**PRKACB**	Renal Water Homeostasis (GO:0003091)	0.007476	0.037531246
**CDC42**	Dendritic Spine Morphogenesis (GO:0060997)	0.007476	0.037531246
**CD44**	Negative Regulation of Intrinsic Apoptotic Signaling Pathway by P53 Class Mediator (GO:1902254)	0.007973	0.037531246
**PRKACB**	Cellular Response to Laminar Fluid Shear Stress (GO:0071499)	0.007973	0.037531246
**KCNC2**	Nitric Oxide Mediated Signal Transduction (GO:0007263)	0.007973	0.037531246
**HSPB1**	Regulation of Endothelial Cell Chemotaxis (GO:2001026)	0.008469	0.038918755
**CDC42**	Establishment of Epithelial Cell Polarity (GO:0090162)	0.008966	0.039219363

**KCNC2**	cGMP-mediated Signaling (GO:0019934)	0.009462	0.039219363
**CD44**	Response to Fibroblast Growth Factor (GO:0071774)	0.009462	0.039219363
**SNAP25**	Import Into Cell (GO:0098657)	0.009462	0.039219363
**KCNC2**	Protein Heterooligomerization (GO:0051291)	0.009957	0.039219363
**CDC42**	Transport Along Microtubule (GO:0010970)	0.009957	0.039219363
**PRKACB**	Multicellular Organismal-Level Water Homeostasis (GO:0050891)	0.009957	0.039219363
**CD44**	Negative Regulation of Signal Transduction by P53 Class Mediator (GO:1901797)	0.010453	0.039556529
**HSPB1**	Regulation of Oxidative Stress-Induced Intrinsic Apoptotic Signaling Pathway (GO:1902175)	0.010453	0.039556529
**CDC42**	Modulation by Host of Viral Process (GO:0044788)	0.010948	0.040154948
**CDC42**	Positive Regulation of Lamellipodium Assembly (GO:0010592)	0.011443	0.040154948
**CD44**	Regulation of Heterotypic Cell-Cell Adhesion (GO:0034114)	0.011443	0.040154948
**HSPB1**	Negative Regulation of Oxidative Stress-Induced Intrinsic Apoptotic Signaling Pathway (GO:1902176)	0.011443	0.040154948
**CDC42**	Macrophage Differentiation (GO:0030225)	0.011938	0.040473191
**CD44**	Negative Regulation of Intrinsic Apoptotic Signaling Pathway in Response to DNA Damage (GO:1902230)	0.012433	0.040473191
**CDC42**	Regulation of Actin Filament Bundle Assembly (GO:0032231)	0.012927	0.040473191

**CDC42**	Dendritic Spine Organization (GO:0097061)	0.012927	0.040473191
**CDC42**	Positive Regulation of Filopodium Assembly (GO:0051491)	0.012927	0.040473191
**CDC42**	Phagocytosis, Engulfment (GO:0006911)	0.013421	0.040473191
**CDC42**	Plasma Membrane Invagination (GO:0099024)	0.013421	0.040473191
**CD44**	Wound Healing, Spreading of Cells (GO:0044319)	0.013421	0.040473191
**CDC42**	Wnt Signaling Pathway, Planar Cell Polarity Pathway (GO:0060071)	0.013421	0.040473191
**HSPB1**	Vascular Endothelial Growth Factor Receptor Signaling Pathway (GO:0048010)	0.014409	0.042783191
**HSPB1;CD44**	Negative Regulation of Programmed Cell Death (GO:0043069)	0.014795	0.042927681
**CD44**	Regulation of Cell Morphogenesis (GO:0022604)	0.014902	0.042927681
**CDC42**	Positive Regulation of Lamellipodium Organization (GO:1902745)	0.015396	0.043063172
**HSPB1**	Regulation of Protein Modification Process (GO:0031399)	0.015396	0.043063172
**CDC42**	Regulation of Lamellipodium Assembly (GO:0010591)	0.015889	0.043807432
**SNAP25**	Signal Release From Synapse (GO:0099643)	0.016382	0.044101935
**CD44**	Regulation of DNA Damage Response, Signal Transduction by P53 Class Mediator (GO:0043516)	0.016874	0.044101935
**HSPB1**	Platelet Aggregation (GO:0070527)	0.017367	0.044101935

**CDC42**	Dendrite Morphogenesis (GO:0048813)	0.017367	0.044101935
**CDC42**	Positive Regulation of Substrate Adhesion-Dependent Cell Spreading (GO:1900026)	0.017367	0.044101935
**SNAP25**	Neurotransmitter Secretion (GO:0007269)	0.017367	0.044101935
**CDC42**	Establishment or Maintenance of Epithelial Cell Apical/Basal Polarity (GO:0045197)	0.017859	0.044188936
**HSPB1**	Cellular Response to Vascular Endothelial Growth Factor Stimulus (GO:0035924)	0.017859	0.044188936
**HSPB1**	Anterograde Axonal Transport (GO:0008089)	0.018351	0.044271052
**GFAP**	Regulation of Cellular Component Biogenesis (GO:0044087)	0.018351	0.044271052
**CDC42**	Regulation of Filopodium Assembly (GO:0051489)	0.019825	0.044839635
**CDC42**	Positive Regulation of Cytokinesis (GO:0032467)	0.019825	0.044839635
**CDC42**	Organelle Transport Along Microtubule (GO:0072384)	0.020316	0.044839635
**HSPB1**	Negative Regulation of Kinase Activity (GO:0033673)	0.020316	0.044839635
**CD44**	Connective Tissue Development (GO:0061448)	0.020316	0.044839635
**SNAP25**	Synaptic Vesicle Exocytosis (GO:0016079)	0.021298	0.044839635
**CDC42**	Positive Regulation of Stress Fiber Assembly (GO:0051496)	0.021298	0.044839635
**CDC42**	Non-Canonical Wnt Signaling Pathway (GO:0035567)	0.021298	0.044839635

**SNAP25**	Regulated Exocytosis (GO:0045055)	0.021788	0.044839635
**CDC42**	Positive Regulation of Cell Division (GO:0051781)	0.021788	0.044839635
**CDC42**	Substantia Nigra Development (GO:0021762)	0.021788	0.044839635
**SNAP25**	Synaptic Vesicle Cycle (GO:0099504)	0.022278	0.044839635
**SERPINH1**	Collagen Fibril Organization (GO:0030199)	0.022278	0.044839635
**HSPB1;CD44**	Negative Regulation of Apoptotic Process (GO:0043066)	0.022326	0.044839635
**CDC42**	Mononuclear Cell Differentiation (GO:1903131)	0.022768	0.044839635
**HSPB1**	Homotypic Cell-Cell Adhesion (GO:0034109)	0.022768	0.044839635
**HSPB1**	Positive Regulation of Chemotaxis (GO:0050921)	0.022768	0.044839635
**SNAP25**	Neurotransmitter Transport (GO:0006836)	0.022768	0.044839635
**CD44**	Positive Regulation of Leukocyte Cell-Cell Adhesion (GO:1903039)	0.023748	0.046295772
**PRKACB**	Negative Regulation of TORC1 Signaling (GO:1904262)	0.024237	0.046777161
**HSPB1**	Positive Regulation of Blood Vessel Endothelial Cell Migration (GO:0043536)	0.024726	0.046785374
**CD44**	Positive Regulation of Cell-Cell Adhesion (GO:0022409)	0.024726	0.046785374
**CDC42**	Positive Regulation of Actin Filament Bundle Assembly (GO:0032233)	0.025215	0.047247161

**CDC42**	Regulation of Substrate Adhesion-Dependent Cell Spreading (GO:1900024)	0.025703	0.047699659
**SNAP25**	Regulation of Peptide Hormone Secretion (GO:0090276)	0.027168	0.049831755
**HSPB1**	Positive Regulation of Interleukin-1 Beta Production (GO:0032731)	0.027656	0.049831755
**CD44**	Cellular Response to Fibroblast Growth Factor Stimulus (GO:0044344)	0.027656	0.049831755
**CD44**	Cartilage Development (GO:0051216)	0.028143	0.049831755
**HSPB1**	Regulation of Blood Vessel Endothelial Cell Migration (GO:0043535)	0.028143	0.049831755
**CDC42**	Negative Regulation of Protein-Containing Complex Assembly (GO:0031333)	0.029118	0.051015086
**HSPB1**	Negative Regulation of Intrinsic Apoptotic Signaling Pathway (GO:2001243)	0.029605	0.051015086
**HSPB1**	Negative Regulation of Protein Kinase Activity (GO:0006469)	0.029605	0.051015086
**HSPB1**	Positive Regulation of Interleukin-1 Production (GO:0032732)	0.031064	0.053056074
**HSPB1**	Regulation of Phosphorylation (GO:0042325)	0.032036	0.054235868
**KCNC2**	Action Potential (GO:0001508)	0.033492	0.056046893
**CDC42**	Cell Junction Organization (GO:0034330)	0.033977	0.056046893
**HSPB1**	Regulation of Translational Initiation (GO:0006446)	0.033977	0.056046893
**PRKACB**	Negative Regulation of TOR Signaling (GO:0032007)	0.034461	0.056364663

**CDC42**	Phagocytosis (GO:0006909)	0.034946	0.056676737
**GFAP**	Intermediate Filament Organization (GO:0045109)	0.03543	0.056983258
**CDC42**	Positive Regulation of Cell-Substrate Adhesion (GO:0010811)	0.035914	0.057284363
**CDC42**	Myeloid Leukocyte Differentiation (GO:0002573)	0.036398	0.057404157
**CDC42**	Regulation of Stress Fiber Assembly (GO:0051492)	0.036881	0.057404157
**CDC42**	Regulation of Plasma Membrane Bounded Cell Projection Assembly (GO:0120032)	0.036881	0.057404157
**CDC42**	Positive Regulation of Growth (GO:0045927)	0.038814	0.059570156
**CDC42**	Negative Regulation of Cellular Component Organization (GO:0051129)	0.039296	0.059570156
**CDC42**	Regulation of Cytokinesis (GO:0032465)	0.039778	0.059570156
**HSPB1**	Regulation of Catabolic Process (GO:0009894)	0.039778	0.059570156
**HSPB1**	Regulation of Interleukin-1 Beta Production (GO:0032651)	0.04026	0.059570156
**HSPB1**	Positive Regulation of Tumor Necrosis Factor Production (GO:0032760)	0.04026	0.059570156
**HSPB1**	Negative Regulation of Protein Phosphorylation (GO:0001933)	0.040742	0.059570156
**HSPB1**	Regulation of Protein Kinase Activity (GO:0045859)	0.040742	0.059570156
**HSPB1**	Positive Regulation of Tumor Necrosis Factor Superfamily Cytokine Production (GO:1903557)	0.041705	0.060068107
**CDC42**	Positive Regulation of Endocytosis (GO:0045807)	0.041705	0.060068107

**CDC42**	Integrin-Mediated Signaling Pathway (GO:0007229)	0.042187	0.060311084
**HSPB1**	Positive Regulation of Endothelial Cell Migration (GO:0010595)	0.043148	0.061232428
**CDC42**	Positive Regulation of Plasma Membrane Bounded Cell Projection Assembly (GO:0120034)	0.044109	0.062139097
**SNAP25**	Regulation of Insulin Secretion (GO:0050796)	0.045549	0.063702256
**HSPB1;CD44**	Regulation of Apoptotic Process (GO:0042981)	0.046163	0.064097023
**CDC42**	Regulation of Actin Filament-Based Process (GO:0032970)	0.046986	0.064774211
**PRKACB**	Regulation of TORC1 Signaling (GO:1903432)	0.047465	0.064970204
**SNAP25**	Regulation of Protein Secretion (GO:0050708)	0.048422	0.065813322
**CDC42**	Cell Junction Assembly (GO:0034329)	0.049378	0.06664348
**CDC42**	Regulation of Actin Cytoskeleton Organization (GO:0032956)	0.050811	0.06810065
**CD44**	Cell-Matrix Adhesion (GO:0007160)	0.052241	0.069058868
**CDC42**	Positive Regulation of Cell Growth (GO:0030307)	0.052241	0.069058868
**HSPB1**	Positive Regulation of Vasculature Development (GO:1904018)	0.053194	0.069839823
**CD44**	T Cell Activation (GO:0042110)	0.055572	0.072468748
**KCNC2**	Protein Complex Oligomerization (GO:0051259)	0.056522	0.073212431

**CDC42**	Positive Regulation of Cell Cycle Process (GO:0090068)	0.057944	0.074555052
**HSPB1**	Positive Regulation of Angiogenesis (GO:0045766)	0.061257	0.078295067
**KCNC2**	Potassium Ion Transport (GO:0006813)	0.061729	0.078379724
**CDC42**	Golgi Organization (GO:0007030)	0.062201	0.078463004
**GFAP**	Regulation of Protein Catabolic Process (GO:0042176)	0.062673	0.078544934
**HSPB1**	Regulation of Tumor Necrosis Factor Production (GO:0032680)	0.063616	0.079212624
**KCNC2**	Potassium Ion Transmembrane Transport (GO:0071805)	0.06503	0.080453224
**HSPB1**	Regulation of Protein Phosphorylation (GO:0001932)	0.066441	0.08167566
**CDC42**	Regulation of Cytoskeleton Organization (GO:0051493)	0.067381	0.082306689
**KCNC2**	Protein Homooligomerization (GO:0051260)	0.069258	0.084067423
**CDC42**	Regulation of Cell Cycle Process (GO:0010564)	0.070663	0.08523742
**CD44**	Positive Regulation of ERK1 and ERK2 Cascade (GO:0070374)	0.073935	0.088630496
**CDC42**	Neuron Projection Morphogenesis (GO:0048812)	0.0758	0.090297156
**CDC42**	Actin Filament Organization (GO:0007015)	0.076266	0.090297156
**GFAP**	Regulation of Cellular Component Organization (GO:0051128)	0.076732	0.090297156
**CD44**	Skeletal System Development (GO:0001501)	0.077197	0.090297156

**KCNC2**	Metal Ion Transport (GO:0030001)	0.086459	0.100522177
**SERPINH1**	Extracellular Matrix Organization (GO:0030198)	0.087381	0.100985361
**SNAP25**	Anterograde Trans-Synaptic Signaling (GO:0098916)	0.0906	0.104082397
**CDC42**	Endomembrane System Organization (GO:0010256)	0.093809	0.107131106
**HSPB1**	Regulation of Translation (GO:0006417)	0.096095	0.108973285
**PRKACB**	Adenylate Cyclase-Modulating G Protein-Coupled Receptor Signaling Pathway (GO:0007188)	0.096551	0.108973285
**CDC42**	Neuron Projection Development (GO:0031175)	0.098376	0.110386513
**HSPB1**	Regulation of Angiogenesis (GO:0045765)	0.100651	0.112286943
**CDC42**	Regulation of Cell Growth (GO:0001558)	0.105639	0.117003204
**CD44**	Regulation of ERK1 and ERK2 Cascade (GO:0070372)	0.106091	0.117003204
**CDC42**	Positive Regulation of Cell Motility (GO:2000147)	0.117776	0.129152292
**SNAP25**	Chemical Synaptic Transmission (GO:0007268)	0.122678	0.133498355
**CD44**	Inflammatory Response (GO:0006954)	0.123123	0.133498355
**HSPB1**	Regulation of Canonical NF-kappaB Signal Transduction (GO:0043122)	0.127114	0.137055518
**CDC42**	Cellular Component Assembly (GO:0022607)	0.130206	0.139610128

**HSPB1**	Cell Surface Receptor Protein Tyrosine Kinase Signaling Pathway (GO:0007169)	0.138551	0.147736788
**PRKACB**	Phosphorylation (GO:0016310)	0.139425	0.147852072
**HSPB1**	Regulation of Intracellular Signal Transduction (GO:1902531)	0.141171	0.148885529
**KCNC2**	Monoatomic Cation Transmembrane Transport (GO:0098655)	0.142043	0.148990806
**KCNC2**	Inorganic Cation Transmembrane Transport (GO:0098662)	0.143349	0.149548134
**CDC42**	Positive Regulation of Cell Migration (GO:0030335)	0.159313	0.165308174
**CD44**	Positive Regulation of MAPK Cascade (GO:0043410)	0.166981	0.172338921
**PRKACB**	Protein Phosphorylation (GO:0006468)	0.171637	0.17620141
**KCNC2**	Cellular Response to Oxygen-Containing Compound (GO:1901701)	0.176269	0.179999103
**CDC42**	Organelle Organization (GO:0006996)	0.198261	0.201391487
**CDC42**	Regulation of Cell Migration (GO:0030334)	0.215312	0.217566758
**PRKACB**	Protein Modification Process (GO:0036211)	0.241448	0.242705694
**CDC42**	Positive Regulation of Cellular Process (GO:0048522)	0.270948	0.270948067

Table S3. Presenting the genes with KEGG enrichment pathways

**Genes**	**Term**	**P-value**	**Adjusted P-value**
**CDC42;CDKN1A;PRKACB;CD44**	Proteoglycans in cancer	2.14E-06	2.60E-04
**CDC42;CDKN1A;PRKACB**	Viral carcinogenesis	1.17E-04	0.007097087
**CDC42;HSPB1;PRKACB**	MAPK signaling pathway	3.50E-04	0.010456638

**CDC42;HSPB1**	VEGF signaling pathway	3.79E-04	0.010456638
**CDC42;CDKN1A;PRKACB**	Human papillomavirus infection	4.94E-04	0.010456638
**CDC42;CDKN1A**	Renal cell carcinoma	5.19E-04	0.010456638
**CDC42;CDKN1A**	Pancreatic cancer	6.29E-04	0.01086805
**SNAP25;PRKACB**	Insulin secretion	8.04E-04	0.012163097
**CDC42;PRKACB**	GnRH signaling pathway	9.39E-04	0.012630901
**HSPB1;PRKACB**	Amoebiasis	0.001128495	0.013396853
**CDKN1A;PRKACB**	Parathyroid hormone synthesis, secretion and action	0.001217896	0.013396853
**CDC42;CDKN1A;PRKACB**	Pathways in cancer	0.00194272	0.019589093
**CDKN1A;PRKACB**	Oxytocin signaling pathway	0.002545458	0.022281668
**CDKN1A;PRKACB**	Cushing syndrome	0.002578044	0.022281668
**CDKN1A;GFAP**	JAK-STAT signaling pathway	0.002811692	0.022680979
**CDC42;PRKACB**	Tight junction	0.003055003	0.023103458
**CDC42;PRKACB**	Chemokine signaling pathway	0.003921793	0.027913942
**CDKN1A;CD44**	Epstein-Barr virus infection	0.004330505	0.029110617
**CDKN1A;PRKACB**	Human T-cell leukemia virus 1 infection	0.005068989	0.032281453
**CDKN1A;PRKACB**	Human cytomegalovirus infection	0.005342638	0.032322961
**CDC42;PRKACB**	Ras signaling pathway	0.005670401	0.032672311
**CDC42;CD44**	Shigellosis	0.006353194	0.034942567
**CDKN1A;CD44**	MicroRNAs in cancer	0.009926386	0.052221421
**CDKN1A**	Thyroid cancer	0.018350695	0.086179746
**CDKN1A**	Bladder cancer	0.020316294	0.086179746
**PRKACB**	Vasopressin-regulated water reabsorption	0.021788169	0.086179746
**PRKACB**	Cocaine addiction	0.024236871	0.086179746
**PRKACB**	Vibrio cholerae infection	0.024725949	0.086179746
**PRKACB**	Ovarian steroidogenesis	0.025214806	0.086179746
**PRKACB**	Endocrine and other factor-regulated calcium reabsorption	0.026191859	0.086179746
**PRKACB**	Regulation of lipolysis in adipocytes	0.027168031	0.086179746
**PRKACB**	Hedgehog signaling pathway	0.027655787	0.086179746
**CDKN1A**	Endometrial cancer	0.028630638	0.086179746
**CDKN1A**	Basal cell carcinoma	0.031063919	0.086179746
**PRKACB**	Cortisol synthesis and secretion	0.032035694	0.086179746
**PRKACB**	Long-term potentiation	0.033006592	0.086179746
**PRKACB**	Amphetamine addiction	0.033976614	0.086179746
**PRKACB**	Renin secretion	0.033976614	0.086179746

**CDC42**	Epithelial cell signaling in Helicobacter pylori infection	0.034461296	0.086179746
**CDC42**	Adherens junction	0.03494576	0.086179746
**CDKN1A**	Melanoma	0.035430005	0.086179746
**CDKN1A**	Non-small cell lung cancer	0.035430005	0.086179746
**CDKN1A**	p53 signaling pathway	0.035914031	0.086179746
**PRKACB**	Thyroid hormone synthesis	0.036881427	0.086179746
**CDKN1A**	Glioma	0.036881427	0.086179746
**CDKN1A**	Chronic myeloid leukemia	0.037364798	0.086179746
**PRKACB**	Gastric acid secretion	0.037364798	0.086179746
**CDC42**	Bacterial invasion of epithelial cells	0.03784795	0.086179746
**SNAP25**	Synaptic vesicle cycle	0.038330884	0.086179746
**CDKN1A**	ErbB signaling pathway	0.041705318	0.086179746
**PRKACB**	Taste transduction	0.042186509	0.086179746
**CDKN1A**	Colorectal cancer	0.042186509	0.086179746
**CD44**	ECM-receptor interaction	0.043148239	0.086179746
**PRKACB**	Gap junction	0.043148239	0.086179746
**PRKACB**	GABAergic synapse	0.043628778	0.086179746
**PRKACB**	Bile secretion	0.0441091	0.086179746
**PRKACB**	Morphine addiction	0.044589205	0.086179746
**CDKN1A**	Small cell lung cancer	0.045069092	0.086179746
**PRKACB**	Salivary secretion	0.045548763	0.086179746
**PRKACB**	Dilated cardiomyopathy	0.046986474	0.086179746
**PRKACB**	Circadian entrainment	0.047465278	0.086179746
**CDC42**	Fc gamma R-mediated phagocytosis	0.047465278	0.086179746
**CDKN1A**	Prostate cancer	0.047465278	0.086179746
**PRKACB**	Aldosterone synthesis and secretion	0.047943866	0.086179746
**PRKACB**	Inflammatory mediator regulation of TRP channels	0.047943866	0.086179746
**CD44**	Hematopoietic cell lineage	0.048422237	0.086179746
**CDC42**	AGE-RAGE signaling pathway in diabetic complications	0.048900391	0.086179746
**PRKACB**	Progesterone-mediated oocyte maturation	0.048900391	0.086179746
**PRKACB**	Melanogenesis	0.049378329	0.086179746
**PRKACB**	Longevity regulating pathway	0.049856052	0.086179746
**CDC42**	T cell receptor signaling pathway	0.050810848	0.086593135
**PRKACB**	Glucagon signaling pathway	0.052241423	0.087327255
**CDKN1A**	HIF-1 signaling pathway	0.053194062	0.087327255
**PRKACB**	Serotonergic synapse	0.055096754	0.087327255
**PRKACB**	Cholinergic synapse	0.055096754	0.087327255

**PRKACB**	Glutamatergic synapse	0.055571889	0.087327255
**CDC42**	Leukocyte transendothelial migration	0.055571889	0.087327255
**PRKACB**	Growth hormone synthesis, secretion and action	0.057944341	0.087384125
**CDC42**	Neurotrophin signaling pathway	0.057944341	0.087384125
**PRKACB**	Thyroid hormone signaling pathway	0.058891819	0.087384125
**CDKN1A**	Cell cycle	0.060311428	0.087384125
**PRKACB**	Platelet activation	0.060311428	0.087384125
**PRKACB**	Oocyte meiosis	0.06267316	0.087384125
**PRKACB**	Relaxin signaling pathway	0.06267316	0.087384125
**CDKN1A**	FoxO signaling pathway	0.063616356	0.087384125
**PRKACB**	Dopaminergic synapse	0.064087634	0.087384125
**PRKACB**	Vascular smooth muscle contraction	0.064558698	0.087384125
**PRKACB**	Apelin signaling pathway	0.066440822	0.087384125
**PRKACB**	Autophagy	0.066440822	0.087384125
**CDC42**	Yersinia infection	0.066440822	0.087384125
**PRKACB**	Estrogen signaling pathway	0.066440822	0.087384125
**PRKACB**	Insulin signaling pathway	0.066440822	0.087384125
**CDKN1A**	Breast cancer	0.07113123	0.091423329
**PRKACB**	Retrograde endocannabinoid signaling	0.071599103	0.091423329
**CDKN1A**	Gastric cancer	0.072066763	0.091423329
**PRKACB**	Adrenergic signaling in cardiomyocytes	0.072534211	0.091423329
**CDC42**	Non-alcoholic fatty liver disease	0.074868275	0.092644957
**CDKN1A**	Cellular senescence	0.075334453	0.092644957
**CDKN1A**	Hepatitis C	0.075800419	0.092644957
**CDKN1A**	Hepatitis B	0.078127083	0.09453377
**PRKACB**	Wnt signaling pathway	0.079984617	0.095823155
**CDKN1A**	Hepatocellular carcinoma	0.08091212	0.095983986
**CDC42**	Axon guidance	0.087381116	0.102651602
**PRKACB**	Alcoholism	0.089221858	0.1038062
**CDKN1A**	Transcriptional misregulation in cancer	0.091976707	0.105515559
**CDKN1A**	Kaposi sarcoma-associated herpesvirus infection	0.092435118	0.105515559
**CDC42**	Pathogenic Escherichia coli infection	0.094266683	0.106600641
**CDC42**	Focal adhesion	0.09609492	0.107661901
**CDC42**	Rap1 signaling pathway	0.100196316	0.111227103
**CDC42**	Lipid and atherosclerosis	0.102467625	0.112171248

**PRKACB**	cAMP signaling pathway	0.102921267	0.112171248
**CDC42**	Regulation of actin cytoskeleton	0.103827932	0.112171248
**PRKACB**	Thermogenesis	0.110151531	0.11794987
**PRKACB**	Chemical carcinogenesis	0.113298247	0.119681583
**PRKACB**	Calcium signaling pathway	0.113746959	0.119681583
**CDC42**	Salmonella infection	0.117776183	0.121802719
**PRKACB**	Parkinson disease	0.117776183	0.121802719
**CDC42**	Endocytosis	0.11911559	0.122143952
**PRKACB**	Prion disease	0.128440307	0.130598967
**CDKN1A**	PI3K-Akt signaling pathway	0.163580733	0.164943906
**PRKACB**	Olfactory transduction	0.199489954	0.199489954
